# Multi‐Stimuli‐Responsive Hydrogen‐Bonded Organic Frameworks Nanocarriers Enable Targeted Fungicide Release and Plant Immune Regulation

**DOI:** 10.1002/advs.75922

**Published:** 2026-06-04

**Authors:** Guangming Ma, Ying Zou, Yao Wang, Huiyan Li, Jinlin Li, Tianfu Liu, Xiaohong Pan

**Affiliations:** ^1^ State Key Laboratory of Agricultural and Forestry Biosecurity & Key Lab of Biopesticide and Chemical Biology College of Plant Protection Ministry of Education & Ministerial and Provincial Joint Innovation Centre For Safety Production of Cross‐Strait Crops Fujian Agriculture and Forestry University Fuzhou Fujian P. R. China; ^2^ State Key Laboratory of Structural Chemistry Fujian Institute of Research on the Structure of Matter Chinese Academy of Sciences Fuzhou Fujian P. R. China

**Keywords:** controlled release, hydrogen‐bonded organic frameworks, multi‐stimuli‐responsive release, plant immune activation, precision agriculture

## Abstract

Precision plant protection requires carriers that couple crystalline order with context‐responsive release. Hydrogen‐bonded organic frameworks (HOFs) offer ordered, metal‐free porosity and programmable host–guest interactions to realize this goal. We therefore built a multi‐stimuli‐responsive system (PYR@PFC‐1‐F@HACC) using the fluorinated HOF PFC‐1‐F as a pesticide carrier and label‐free in planta fluorescent marker. Pyraclostrobin (PYR) was encapsulated in PFC‐1‐F with ∼30 wt.% loading, and the resulting composite was surface‐coated with chitosan quaternary ammonium salt (HACC). The HACC shell imparts leaf adhesion and biointerface compatibility, thereby enabling enzyme/reactive oxygen species (ROS)/pH‐triggered, site‐specific release of the fungicide while suppressing leakage near neutrality. Under pathogen‐mimicking conditions, the composite shows hierarchically programmed, multi‐stimuli‐responsive release with diffusion‐dominated kinetics. Antifungal activity follows a dual pathway‐membrane disruption together with ROS‐associated oxidative stress‐accompanied by host‐defense remodeling. In planta, the platform curtails lesion development, reduces pathogen adhesion/invasion, and exhibits robust foliar retention with acropetal transport. Standard biosafety assays show no significant adverse effects at working doses on peanut seed germination, zebrafish, earthworms, or silkworms. Overall, this study delivers a generalizable, sustainable nanocarrier for precision management of soil‐borne crop diseases.

## Graphical abstract

A fluorinated hydrogen‐bonded organic framework nanocarrier (PFC‐1‐F) loaded with PYR and coated with HACC enables multi‐stimuli‐responsive (enzyme/ROS/pH) release at infection sites. Exhibiting targeted antifungal activity against *Sclerotium rolfsii*, modulating plant antioxidant defense, and demonstrating favorable biosafety across plant, soil, and aquatic organisms, this system provides a sustainable platform for precision disease management.

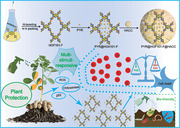

## 1 Introduction

Peanut (*Arachis hypogaea L*.) is a globally traded legume valued for its edible oil and protein content [1]. China is one of the world's leading peanut producer, with approximately 5 million hectares under cultivation and an annual output of about 17 million tonnes accounting for nearly 40% of global production [2]. In tropical and subtropical production systems, disease pressure represents a major constraint on peanut yield, often exceeding the impacts of drought or low‐input management—with stem rot (white mold) caused by *Sclerotium rolfsii* a principal threat; epidemics commonly cut yields by 10–50% and incur substantial regional economic losses [3]. The pathogen colonizes basal stems 1 month after sowing, secretes organic acids that drive cell necrosis and maceration, and ultimately causes lodging and death; white mycelia and sclerotia are diagnostic of infection [4, 5]. Although chemical fungicides remain the mainstay, field control is hindered by the pathogen's broad host range and soil persistence [6], while long‐term use raises concerns about environmental contamination, resistance development, and effects on non‐target organisms [7]. Biological agents (e.g., *Trichoderma* spp.) are attractive but often show inconsistent field efficacy, short shelf life, and environmental fluctuations [8, 9]. Hence, a carrier that can concentrate actives at infection sites and release them on cue is urgently needed.

From a materials standpoint, practical deployment also imposes constraints: formulations must be rainfast on hydrophobic foliage, begin to plant surfaces, safe across the life cycle, and manufacturable at scale without batch drift. Nanotechnology can encode solubility, deposition, and spatiotemporal release [10], nano‐enabled carriers improve photostability, leaf retention, and cuticular penetration, and support sustained or stimuli‐responsive delivery—raising efficacy while lowering off‐target loads [11, 12, 13, 14]. Hydrogen‐bonded organic frameworks (HOFs) address these needs as crystalline, metal‐free networks assembled from small organic linkers via directional H‐bonding and ancillary π–π/van der Waals interactions [15]. Their ordered microporosity supports efficient loading, while the absence of metal nodes improves biocompatibility and avoids metallic ecotoxicity [16, 17, 18]. Because linker chemistry is programmable, F‐substitution strategy can be tuned to raise pesticide stability and load factor by regulate HOFs–pesticide interactions, while HOF lattices can be designed to respond to infection‐site cues for logic‐gated release. Together, these attributes position HOFs as a materials‐driven solution for next‐generation, multi‐stimuli‐responsive agrochemical delivery.

Herein, we apply this concept for pyraclostrobin (PYR)‐a broad‐spectrum strobilurin targeting mitochondrial complex III whose field performance is limited by strong hydrophobicity, UV photodegradation, and aquatic toxicity concerns [19, 20, 21, 22, 23, 24]. We engineer a crystalline nanoplatform that integrates a fluorinated HOF core (PFC‐1‐F also termed HOF‐101‐F) with HACC. Fluorine‐rich channel walls provide C─H···F contacts and hydrophobic/van‐der‐Waals interactions that increase PYR affinity and suppress premature leakage, while the HACC coating supplies leaf‐surface adhesion, enhanced biointerface association with fungal/plant surfaces, and chitosanase susceptibility for enzyme‐triggered erosion [25, 26]. Together this fully organic architecture couples ordered microporosity with multi‐cue logic (enzyme/pH/ROS) to enable on‐demand release at pathogen‐associated interfaces, translating into effective suppression of peanut stem rot with a favorable safety profile.

## 2 Results and Discussion

### 2.1 Structural Characterization of PFC‐1‐F

**FIGURE 1:**
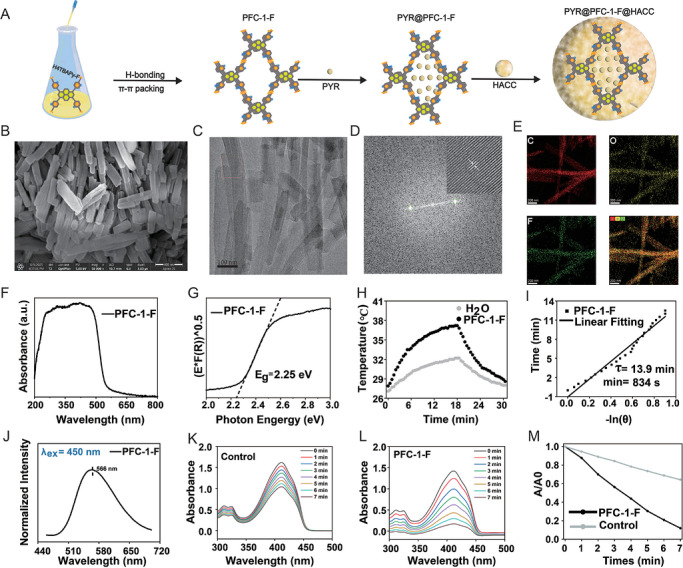
Structural characterization of PFC‐1‐F. (A) Schematic illustration of the self‐assembly and composite formation process, H_4_TBAPy‐F monomers assemble into PFC‐1‐F through hydrogen bonding and π–π stacking, followed by PYR encapsulation and HACC coating. (B) SEM image of PFC‐1‐F showing uniform rod‐like morphology. (C) TEM image revealing the ordered internal framework. (D) High‐resolution TEM image with corresponding lattice fringes confirming crystalline order. (E) Elemental mapping images of C, O, and F, verifying homogeneous distribution of fluorine functionalities. (F) UV–Vis absorption spectrum of PFC‐1‐F. (G) Tauc plot analysis estimating an optical bandgap of 2.25 eV. (H) Photothermal heating profile of PFC‐1‐F suspension under 450 nm LED irradiation (200 mW/cm^2^) compared with water. (I) Linear fitting of cooling curve, yielding a photothermal conversion efficiency of ∼16%. (J) Photoluminescence spectrum of PFC‐1‐F showing an emission peak centered at 566 nm upon excitation at 450 nm. (K–M) ROS generation analysis using DPBF as a probe: (K) Time‐dependent absorbance changes under visible‐light irradiation, (L) Corresponding absorption spectra, and (M) Kinetic fitting curves confirming efficient ROS production.

Self assembly of tetramethyl 4, 4', 4'', 4'''‐(pyrene‐1, 3, 6, 8‐tetrayl)tetrakis(2‐fluorobenzoate) (H_4_TBAPy‐F) through complementary hydrogen bonds yields a 2D‐layered packing structure (PFC‐1‐F) (Figure 1A). Scanning electron microscopy (SEM) and high‐resolution transmission electron microscopy (HRTEM) confirmed that the resulting nanocrystals exhibited a uniform rod‐like morphology with smooth surfaces and well‐defined lattice features (Figure 1B–D). Elemental mapping demonstrated the homogeneous distribution of carbon, oxygen, and fluorine within the crystals (Figure 1E). These structural attributes endow PFC‐1‐F with a robust crystalline scaffold with high porosity and functional tunability, laying the foundation for subsequent fungicide encapsulation and delivery.

Optical measurements further revealed that PFC‐1‐F possesses strong visible‐light activity. UV–vis absorption spectra revealed strong absorption in the 280–500 nm range (Figure 1F), with an estimated optical bandgap of 2.25 eV determined from Tauc plot analysis (Figure 1G). Photoluminescence spectra exhibited a characteristic emission peak centered at 566 nm upon excitation at 450 nm (Figure 1J). PFC‐1‐F displayed excellent photothermal performance. Upon 450 nm LED irradiation (200 mW/cm^2^), the suspension temperature rapidly increased to 39.5°C within 15 min, significantly higher than the water control (Figure 1H). Kinetic fitting revealed a photothermal conversion efficiency of approximately 16% (Figure 1I), confirming efficient light‐to‐heat transformation. Moreover, the ROS generation capacity of PFC‐1‐F was examined using 1,3‐Diphenylisobenzofuran (DPBF) as a probe. Under visible‐light irradiation, a rapid and pronounced decline in DPBF absorbance was observed (Figure 1K–M), with first‐order kinetic fitting confirming efficient ROS production. Based on this stable, visible‐light‐responsive scaffold, we next load PYR and engineer a cationic HACC shell to construct PYR@PFC‐1‐F@HACC for multi‐stimuli‐responsive delivery.

### 2.2 Material Design and Composite Formation

To overcome the poor aqueous solubility and short field persistence of PYR [27], we constructed a hierarchical composite by loading PYR into PFC‐1‐F and then electrostatically depositing HACC onto its surface. PFC‐1‐F provides fluorine‐rich channels for loading PYR, whereas HACC contributes adhesion and enzyme/pH sensitivity to improve on‐leaf retention and stimuli‐responsive release.

**FIGURE 2:**
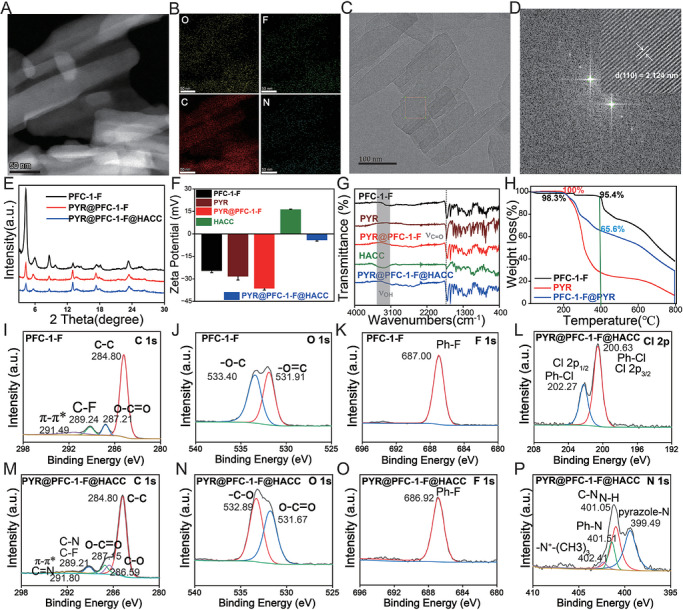
Structural characterization of PYR@PFC‐1‐F and PYR@PFC‐1‐F@HACC composites. (A) High‐resolution TEM image of PYR@PFC‐1‐F showing rod‐like morphology with ordered crystalline features. (B) Elemental mapping images of O, F, C, and N in PYR@PFC‐1‐F, confirming uniform distribution. (C) TEM image of PYR@PFC‐1‐F highlighting the ordered porous framework. (D) HRTEM lattice image and FFT analysis of PYR@PFC‐1‐F, with interplanar spacing indexed to the (110) plane (*d* = 2.124 nm). (E) Powder XRD patterns of PFC‐1‐F, PYR, PYR@PFC‐1‐F, and PYR@PFC‐1‐F@HACC, demonstrating retention of framework crystallinity after drug loading and polymer coating. (F) Zeta potential measurements of individual and composite materials, showing stepwise evolution of surface charge during assembly. (G) FTIR spectra of PFC‐1‐F, PYR, HACC, PYR@PFC‐1‐F, and PYR@PFC‐1‐F@HACC, confirming the coexistence of characteristic vibrational bands from each component. (H) TGA curves of PFC‐1‐F, PYR, and PYR@PFC‐1‐F, highlighting enhanced thermal stability. (I–K) High‐resolution XPS spectra of PFC‐1‐F (C 1s, O 1s, F 1s). (L‐P) High‐resolution XPS spectra of PYR@PFC‐1‐F@HACC (Cl 2p, C 1s, O 1s, F 1s, N 1s).

TEM and HRTEM analyses revealed that PYR@PFC‐1‐F preserved the rod‐like crystalline morphology with visible lattice fringes (Figure 2A‐D), while elemental mapping confirmed the uniform distribution of C, N, O, and F, validating the successful incorporation of PYR. XRD patterns (Figure 2E) further demonstrated that the crystalline framework of PFC‐1‐F remained intact after drug loading and polymer coating, indicating pore occupancy without lattice collapse. Such retention of crystallinity is crucial for maintaining a high surface area and facilitating long‐term controlled release. Surface charge analysis revealed the electrostatic basis of composite assembly (Figure 2F). Pristine PFC‐1‐F carried a negative potential (∼−25 mV), which further decreased after PYR encapsulation (∼−35 mV), likely due to the occupation of internal pores and increased electron density on the surface. In contrast, HACC exhibited a strongly positive potential (∼+15 mV), enabling its electrostatic adsorption onto the negatively charged PYR@PFC‐1‐F surface. Following coating, the zeta potential of PYR@PFC‐1‐F@HACC shifted to ∼−5 mV, approaching neutrality. Such surface charge modulation plays a critical role in nano‐agrochemical performance, as near‐neutral systems exhibit enhanced adhesion to hydrophobic plant cuticles and improved penetration across biological membranes [28, 29, 30]. Fourier transform infrared spectroscopy (FTIR) provided further validation of composite formation (Figure 2G). PYR exhibited distinct C═O stretching (1715 cm^−1^), while HACC contributed broad O─H/N─H stretching bands at 3312 cm^−1^. Both sets of characteristic peaks were retained in the PYR@PFC‐1‐F@HACC spectrum, confirming the successful integration of drug and polymer. Thermal analysis revealed that free PYR underwent significant degradation at 200°C–300°C, while PYR@PFC‐1‐F displayed improved stability with a delayed onset of weight loss. The addition of HACC further increased thermal stability and residual mass (Figure 2H), underscoring the protective role of both the HOF scaffold and polymer coating in stabilizing active ingredients under harsh agricultural conditions. In addition, the PYR loading in PYR@PFC‐1‐F was quantified by TGA. Because PFC‐1‐F remained essentially constant during the major decomposition interval of free PYR (200°C‐300°C), the additional mass loss of PYR@PFC‐1‐F relative to PFC‐1‐F in this window was attributed to encapsulated PYR. Using the residual masses at the end of this interval, the PYR loading was calculated as 29.8 wt.% (≈30 wt.%). The surface elemental composition of the prepared nanoparticles were analyzed by XPS (Figure 2I‐P). The survey spectrum of the carrier displayed characteristic peaks for C 1s, O 1s, and F 1s, matching the expected composition of PFC‐1‐F. For PYR@PFC‐1‐F@HACC, additional peaks for N 1s and Cl 2p were observed, consistent with the composite's expected elements. High‐resolution XPS analysis, with all binding energies charge‐corrected to C─C (284.80 eV), revealed four major carbon bonds in the C 1s spectrum of PFC‐1‐F: C─C (284.80 eV), O─C═O (287.21 eV), C─F (289.24 eV), and π–π* (291.80 eV). PYR@PFC‐1‐F@HACC showed a distinct N 1s peak at 401.05 eV, assigned to overlapping N─H and C─N groups, N 1s peak at 402.41 eV, assigned to N^+^─(CH_3_)_3_ groups, which can verify the HACC coating [31]. N 1s peaks at 399.49 eV (pyrazole N), 401.51 eV (phenyl N), Cl 2p peaks at 200.63 and 202.27 eV corresponding to Cl 2p_3/2_ and Cl 2p_1/2_, verify the PYR loading. The O 1s peaks at 531.91 eV (─O═C) and 533.40 eV (─O─C) in PFC‐1‐F shift to 531.67 and 532.89 eV in PYR@PFC‐1‐F@HACC and the F 1s peaks at 687.00 eV in PFC‐1‐F shift to 686.92 eV in PYR@PFC‐1‐F@HACC likely due to hydrogen bonding between PFC‐1‐F with PYR and HACC, which increased the electron cloud density around the O/F atoms acting as hydrogen‐bond acceptors and consequently decreased the binding energies of these characteristic peaks.

In summary, the PYR@PFC‐1‐F@HACC composite demonstrates the following key features, preserved crystalline architecture and porosity, high drug‐loading efficiency (∼30%), enhanced thermal and structural stability, and tunable surface charge for improved adhesion and biocompatibility. These attributes collectively establish a structurally stable and agro‐compatible nanoplatform, laying a robust foundation for subsequent multi‐stimuli‐responsive release and in planta antifungal evaluations.

### 2.3 Multi‐Stimuli‐Responsive Release Behavior Under Simulated Pathogenic Microenvironment

**FIGURE 3:**
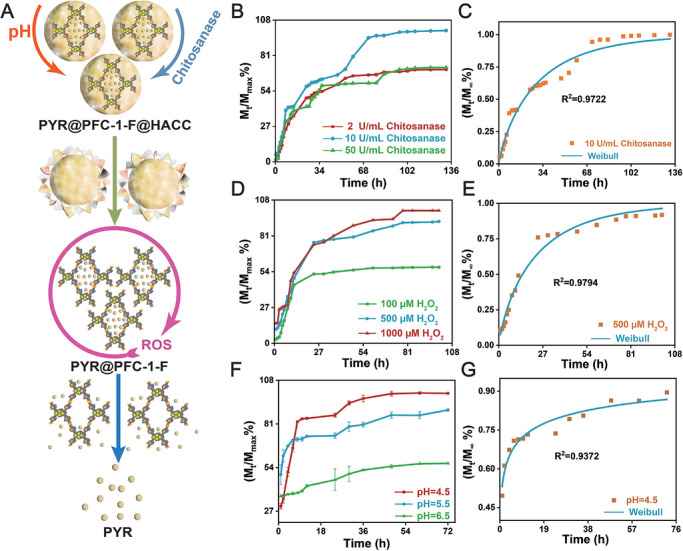
Multi‐stimuli‐responsive release behavior of PYR@PFC‐1‐F@HACC. (A) Schematic illustration of multi‐stimuli‐triggered release pathways under chitosanase, ROS (H_2_O_2_), and acidic pH conditions. (B) Cumulative PYR release under different chitosanase concentrations (2, 10, 50 U/mL). (C) Weibull model fitting of release at 10 U/mL chitosanase (R^2^ = 0.9722). (D) PYR release under different H_2_O_2_ concentrations (100, 500, 1000 µm). (E) Weibull model fitting of release at 500 µm H_2_O_2_ (R^2^ = 0.9794). (F) pH‐responsive PYR release at pH 4.5, 5.5, and 6.5. (G) Weibull model fitting of release kinetics at pH 4.5 (R^2^ = 0.9372).

The pathogenic fungus *S. rolfsii*, responsible for peanut white rot disease, modifies the rhizosphere microecology during infection by secreting extracellular enzymes, inducing oxidative stress, and regulating local pH conditions to facilitate colonization and expansion [32, 33, 34]. Designing nanocarriers that respond to multiple pathogen‐associated stimuli enables precise pesticide release at infection sites, thereby achieving more effective disease control [35, 36]. In this study, the PYR@PFC‐1‐F@HACC nanoplatform was engineered to achieve controlled and site‐specific release through the cooperative roles of the PFC‐1‐F core and HACC outer shell. Under simulated pathogenic microenvironmental conditions, three key triggers—chitosanase, reactive oxygen species (ROS), and acidic pH‐significantly influenced the release behavior (Figure 3A).

As shown in Figure 3B, the system exhibited pronounced enzyme‐responsiveness, with release rates increasing alongside chitosanase concentration. At 10 U/mL, the cumulative release exceeded 88% within 120 h, consistent with extracellular enzyme levels secreted by *S. rolfsii* during root infection [37, 38]. This behavior is attributed to the degradation of β‐(1→4)‐glycosidic bonds in the HACC coating, which exposes the porous HOF core and accelerates fungicide diffusion [39]. The release profiles were well fitted to the Weibull model (R^2^ = 0.9722, Figure 3C), suggesting a mechanism governed by both enzymatic degradation and diffusion, in line with prior reports on enzyme‐responsive pesticide carriers [40, 41].

ROS‐triggered release was also observed under H_2_O_2_ stimulation (Figure 3D). Here, H_2_O_2_ concentrations of 100, 500, and 1000 µm were selected as a graded oxidative‐stress window, 100 µm represents a biologically relevant level commonly reported for pathogen‐induced oxidative bursts, whereas 500–1000 µm were included as upper‐bound or accelerated conditions to clearly demonstrate stimulus‐dependent release within a practical timeframe and to evaluate performance under severe ROS stress. At concentrations of 500 and 1000 µm, the cumulative release reached approximately 82.4% within 72 h, significantly higher than that under low‐ROS conditions. Kinetic fitting yielded an excellent correlation with the Weibull model (R^2^ = 0.9794, Figure 3E). Although we did not directly capture structural evidence of framework degradation, the accelerated release suggests that oxidative stress perturbs the non‐covalent interactions that stabilize the HOF framework, thereby facilitating PYR diffusion. Similar ROS‐responsive behaviors have been reported in HOF‐ and MOF‐based carriers, where oxidative stress modulates carrier stability and drug release [42, 43, 44, 45, 46, 47]. These results indicate that PYR@PFC‐1‐F@HACC possesses intrinsic oxidation sensitivity, which may enhance its antifungal activity in ROS‐rich infection sites.

Local acidification is another characteristic of pathogenic infection, with rhizosphere pH often dropping to 4.5–5.0 during *S. rolfsii* colonization [48, 49]. As shown in Figure 3F, PYR release was markedly accelerated at pH 4.5 (86.7% within 48 h), whereas release remained limited under near‐neutral conditions (pH 6.5). Weibull model fitting confirmed a diffusion‐controlled process (R^2^ = 0.9372, Figure 3G). This pH sensitivity primarily arises from the protonation state of HACC: under acidic conditions, weakened electrostatic interactions and possible swelling of the polymer shell reduce diffusion barriers, thereby accelerating fungicide release. Importantly, the PFC‐1‐F framework maintained structural integrity at pH ≥ 4.5, indicating good stability in mildly acidic environments.

Overall, these three triggers operate on distinct structural levels—enzyme‐induced HACC degradation, ROS‐mediated modulation of noncovalent interactions, and pH‐regulated polymer swelling—thereby forming a hierarchical release cascade. Compared to previously reported single‐stimulus responsive carriers such as pH‐responsive ZIF‐8 or ROS‐sensitive mesoporous silica nanoparticles [50, 51], PYR@PFC‐1‐F@HACC integrates multiple pathogenic cues, achieving site‐specific pesticide release and representing a promising strategy for precision plant disease management.

### 2.4 Optimization of Wettability and in Planta Transport Behavior of the Nanocarrier System

**FIGURE 4:**
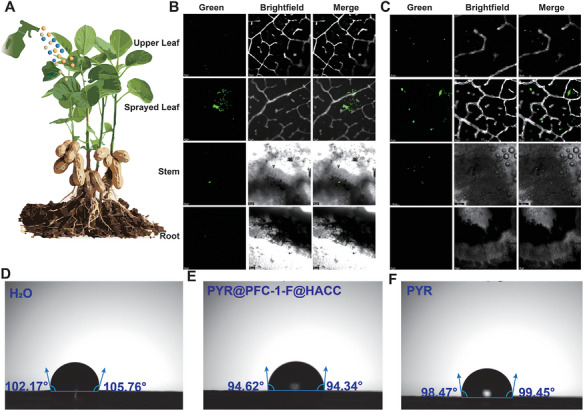
Leaf wettability, foliar retention, and in‐planta transport of PYR@PFC‐1‐F@HACC in peanut. (A) Schematic illustration of foliar spraying of the nanocarrier in peanut. (B, C) Confocal laser scanning microscopy (CLSM) images of peanut tissues collected 12 h (B) and 24 h (C) after foliar application of PYR@PFC‐1‐F@HACC. Columns show the green channel (intrinsic fluorescence of the HOF carrier), bright‐field, and merged images; rows correspond to the upper (unsprayed) leaf, sprayed leaf, stem, and root. (D‐F) Static contact‐angle images on peanut leaves for PYR, PYR@PFC‐1‐F@HACC, and H_2_O (control). The nanocarrier reduces the leaf contact angle relative to water, indicating improved wettability and deposition on the hydrophobic cuticle.

In precision agriculture, the wettability and systemic translocation of nanocarriers are crucial determinants of their targeted delivery efficiency, spatial coverage, and antimicrobial performance against phytopathogens [52]. For soil‐borne diseases like peanut stem rot caused by *S. rolfsii*, which primarily colonizes the rhizosphere and basal stem region, effective foliar‐applied protection benefits from carriers that can penetrate foliar barriers and distribute toward infection‐relevant tissues, enabling a full‐pathway protection strategy from leaf surfaces to internal transport [53]. To evaluate the in planta transport behavior of PYR@PFC‐1‐F@HACC, we leveraged the intrinsic fluorescence of the HOF carrier for label‐free visualization (Figure 4B,C). At 12 h post‐application (Figure 4B), fluorescence signals were mainly confined to the sprayed leaf, indicating strong local retention at the early stage. By 24 h (Figure 4C), fluorescence became detectable in the stem and adjacent unsprayed upper leaves, suggesting upward transport within aerial tissues. In contrast, no obvious signal was observed in roots within this time window, implying that systemic migration under the current foliar application conditions is largely restricted to aboveground tissues. Even without measurable root accumulation, transport toward leaves and stems‐tissues associated with *S. rolfsii* invasion and lesion development‐may still improve coverage of potential infection zones and contribute to pre‐emptive protection at early disease stages. Notably, the self‐fluorescence of the HOF framework enables in situ tracking without external labels, facilitating spatiotemporal analysis of delivery dynamics across tissues.

In parallel, we examined leaf‐surface wettability by contact‐angle measurements (Figure 4D‐F). Compared to free PYR and water, PYR@PFC‐1‐F@HACC exhibited significantly reduced contact angles on peanut leaves, indicative of enhanced hydrophilicity and spreading ability. This improved wettability is expected to promote more uniform and persistent deposition on the hydrophobic cuticle, reduce rain wash‐off, and increase interfacial contact with stomata and/or the cuticular layer. Together, these interfacial advantages provide a practical basis for the strong foliar retention and acropetal redistribution observed in Figure 4B,C under the tested conditions. Consistent with prior reports, tuning surface/interfacial tension is an effective strategy to improve foliar retention and bioavailability of agrochemicals [54]. Overall, these results support the feasibility of this platform for spatiotemporally controlled delivery against complex plant diseases.

### 2.5 Mechanistic Basis for Enhanced Antifungal Activity of PYR@PFC‐1‐F@HACC Against *S. rolfsii*

**FIGURE 5:**
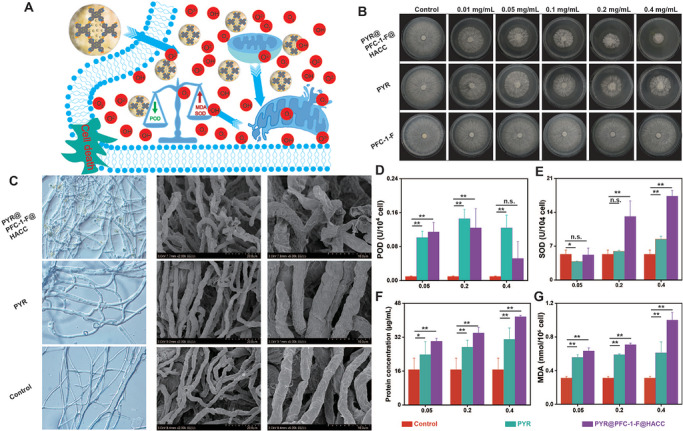
Antifungal activity of PYR@PFC‐1‐F@HACC against *S. rolfsii* and associated cellular responses. (A) Schematic illustration of the antifungal mechanism of PYR@PFC‐1‐F@HACC, depicting ROS scavenging and membrane disruption pathways. (B) Growth inhibition plates of *S. rolfsii* treated with different concentrations (0.01, 0.2, and 0.4 mg/mL) of PYR@PFC‐1‐F@HACC, PYR, and PFC‐1‐F were shown. (C) Optical microscopy and SEM images showing the cellular tissue damage of peanut white mold treated with PYR@PFC‐1‐F@HACC, PYR, and Control. (D–G) Show the effects of different concentrations (0.05, 0.2, and 0.4 mg/mL) of PYR@PFC‐1‐F@HACC and PYR on intracellular oxidative stress markers, specifically including, POD, SOD, Protein concentration, and MDA. Data are means ± SD of triplicate experiments. Data are means ± SD of triplicate experiments. Asterisks (*) indicate a significant difference between sample type and the control according to a Student's *t*‐test (*p < 0.05, **p < 0.01).

To systematically evaluate the antifungal efficacy of PYR@PFC‐1‐F@HACC against *S. rolfsii*, the causal agent of peanut stem rot, a series of dose‐dependent plate inhibition assays was conducted (Figure 5B). The plate results showed clear differences in the inhibitory capacity among the four experimental groups, the control group (Control), the unloaded carrier (PFC‐1‐F), PYR, and the composite formulation (PYR@PFC‐1‐F@HACC). Notably, the PYR@PFC‐1‐F@HACC group exhibited the most significant inhibition, with visibly smaller colony diameters compared to other treatments, indicating superior antifungal activity. In contrast, the Control group displayed dense and vigorous fungal growth, suggesting that *S. rolfsii* proliferates rapidly in the absence of treatment. The PFC‐1‐F group exhibited negligible inhibition, consistent with the material lack of intrinsic antifungal properties. Free PYR showed moderate antifungal activity, forming relatively indistinct inhibition zones. By comparison, the PYR@PFC‐1‐F@HACC group displayed significantly enlarged and well‐defined inhibition zones, implying that the composite material not only stabilizes PYR but also ensures its sustained and localized release around fungal colonies. This enhanced efficacy can be attributed to the highly ordered microporous structure of PFC‐1‐F, which provides excellent drug‐loading and sustained‐release capabilities, and the hydrophilic, cationic HACC coating, which enhances fungus–material interfacial contact. This interfacial effect was further supported by zeta‐potential measurements of *S. rolfsii* hyphal suspensions after treatment (Figure S4). Moreover, the HACC outer shell may prolong contact time at the fungus‐material interface, further enhancing localized antifungal activity.

To further elucidate the underlying antifungal mechanism, the morphological changes of fungal hyphae following treatment were analyzed using optical microscopy and SEM (Figure 5C). At a maximum treatment concentration of 0.4 mg/mL, the Control group exhibited dense, smooth, and well‐branched hyphae under optical microscopy, indicative of healthy growth. In contrast, hyphae treated with free PYR showed slight shrinkage and fuzzy edges, indicating partial inhibition. Remarkably, the PYR@PFC‐1‐F@HACC group exhibited severe morphological abnormalities including hyphal breakage, collapse, and irregular distortion, highlighting the composite ability to disrupt fungal cell integrity. These effects are likely due to the combined contributions of HOF‐enabled sustained local exposure of PYR and HACC‐enhanced biointerface interaction, which together facilitate targeted delivery and penetration of PYR into the fungal cell wall [55]. SEM imaging corroborated these observations. Control group hyphae appeared filamentous, smooth, and continuous. Free PYR treatment caused localized collapse and minor surface roughening. In contrast, PYR@PFC‐1‐F@HACC‐treated hyphae showed extensive surface damage, including severe shrinkage, ruptures, and pore formation, with signs of structural peeling. This confirms that the composite induces fatal damage to fungal cells through a dual mechanism involving mechanical disruption and sustained drug exposure.

**FIGURE 6:**
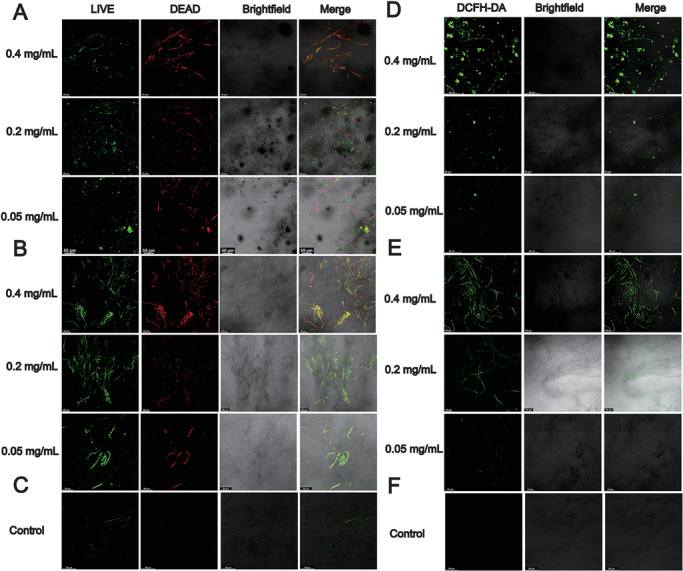
Membrane integrity and intracellular ROS assessment in *S. rolfsii* after treatment with PYR@PFC‐1‐F@HACC and PYR. (A‐C) Live/Dead fluorescence staining of *S. rolfsii* mycelia after treatment with (A) PYR@PFC‐1‐F@HACC, (B) free PYR, and (C) Control at 0.05, 0.2, and 0.4 mg/mL. Red fluorescence indicates dead cells (PI), and green fluorescence indicates live cells (SYTO9). (D‐F) ROS accumulation detected using DCFH‐DA staining after 24 h incubation with (D) PYR@PFC‐1‐F@HACC, (E) free PYR, and (F) Control.

To evaluate post‐treatment viability, Live/Dead double staining was performed (Figure 6A–C). In the PYR@PFC‐1‐F@HACC group, increasing the concentration from 0.05, 0.1, and 0.4 mg/mL led to a clear dose‐dependent enhancement of PI‐positive red signals, indicative of progressive membrane compromise. In contrast, the PYR groups retained predominantly green fluorescence with comparatively weaker PI staining, suggesting less pronounced membrane disruption under the same conditions. Intracellular ROS was further assessed using DCFH‐DA staining (Figure 6D‐F). PYR@PFC‐1‐F@HACC produced the strongest ROS‐associated fluorescence at 0.4 mg/mL, whereas PYR showed weaker signals, and the control remained at near‐background levels. Together, these fluorescence results support that PYR@PFC‐1‐F@HACC induces membrane damage accompanied by elevated oxidative stress, consistent with an oxidative‐stress–associated antifungal killing process.

To further investigate cellular responses, a series of biochemical assays was conducted following 24 h co‐culture of *S. rolfsii* with varying concentrations of the composite (0.05, 0.2, and 0.4 mg/mL). Key physiological markers including superoxide dismutase (SOD), peroxidase (POD), malondialdehyde (MDA), and soluble protein content were quantified to assess oxidative damage and metabolic disruption (Figure 5D‐G). SOD activity increased progressively with rising PYR@PFC‐1‐F@HACC concentration, reflecting a robust antioxidant response. As the first line of defense against superoxide radicals, enhanced SOD activity suggests the material effectively triggers oxidative stress mitigation in fungal cells [56]. In contrast, POD activity peaked at low concentration (0.05 mg/mL) but declined at higher doses (0.4 mg/mL), possibly due to enzyme deactivation or regulatory feedback under excessive stress. This indicates a shift toward SOD‐dominant ROS clearance at higher stress levels. MDA content, an indicator of lipid peroxidation and membrane damage [57], increased steadily with concentration, signifying extensive oxidative injury. Soluble protein content was slightly elevated at low concentrations due to stress‐induced protein synthesis but surged at 0.2 and 0.4 mg/mL, suggesting cytoplasmic metabolic imbalance and membrane leakage. These trends are consistent with the microscopy observations, supporting the hypothesis that ROS accumulation and membrane disruption jointly contribute to cell collapse [58].

### 2.6 Plant‐Level Validation: PYR@PFC‐1‐F@HACC Effectively Alleviates Disease Progression and Activates Systemic Antioxidant Defense Networks

**FIGURE 7:**
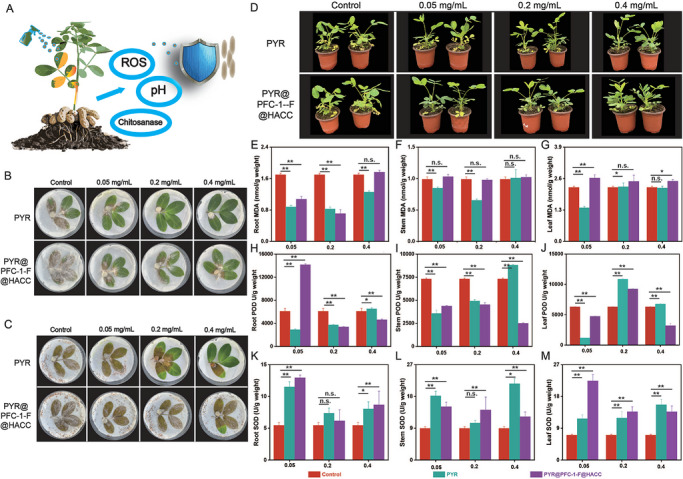
In‐plant antifungal efficacy and host oxidative‐stress responses. (A) Schematic of the multi‐stimuli‐responsive logic of PYR@PFC‐1‐F@HACC (enzyme/ROS/pH) during foliar application and rhizosphere infection. (B, C) Leaf‐disc infection assays inoculated with *S. rolfsii* and treated with water (Control), free PYR, or PYR@PFC‐1‐F@HACC at 0.05, 0.2, 0.4 mg/mL (dose expressed as PYR equivalent). Images recorded at 6 days (B) and 12 days (C) show dose‐dependent suppression by the nanocarrier. (D) Whole‐plant phenotypes at 6 days under the same treatments, illustrating improved disease resistance with the nanocarrier. (E–G) Malondialdehyde (MDA) contents in roots, stems, and leaves after challenge. (H–J) Peroxidase (POD) activities in roots, stems, and leaves. (K–M) Superoxide dismutase (SOD) activities in roots, stems, and leaves. Data are means ± SD of triplicate experiments. Asterisks (*) indicate a significant difference between sample type and the control according to a Student's *t*‐test (*p < 0.05, **p < 0.01).

Guided by these in vitro indications of membrane disruption and ROS accumulation in *S. rolfsii*, we next assessed the in planta antifungal efficacy of PYR@PFC‐1‐F@HACC under realistic exposure conditions. As illustrated schematically in Figure 7A, the antifungal activity of PYR@PFC‐1‐F@HACC is governed by a multi‐stimuli‐responsive release mechanism triggered by pH, ROS, and chitosanase. This intelligent release behavior results in targeted membrane disruption, enhanced ROS accumulation, and activation of host immune responses, ultimately reinforcing the plant's defense system. To validate it in plant efficacy, a simulated infection model was established by inoculating *S rolfsii* at the peanut rhizosphere, followed by foliar application of different treatments, including an untreated control, free PYR, and PYR@PFC‐1‐F@HACC at concentrations of 0.05, 0.2, and 0.4 mg/mL. As shown in Figure 7B,C, lesion progression at 6 and 12 days post‐inoculation was significantly suppressed in all nanocarrier‐treated groups, with the highest concentration (0.4 mg/mL) showing the most pronounced protective effect. In particular, PYR@PFC‐1‐F@HACC‐treated leaves exhibited visibly reduced symptom severity and lighter lesion coloration, suggesting effective inhibition of pathogen colonization and invasion. Quantitative disease‐related analysis further confirmed the superior antifungal performance of the nanocarrier system compared to free PYR. Moreover, whole‐plant phenotypes observed at day 6 (Figure 7D) demonstrated improved disease resistance in plants treated with PYR@PFC‐1‐F@HACC, reinforcing the potential of this multifunctional delivery platform for crop protection.

To elucidate the underlying protective mechanism, we assessed the physiological responses of plants following pathogen challenge and examined the regulatory effects of different treatments. Three key oxidative stress indicators MDA, POD, and SOD were analyzed in roots, stems, and leaves (Figure 7E‐M). MDA, a hallmark marker of lipid peroxidation and membrane damage, was markedly elevated under pathogen stress. However, PYR@PFC‐1‐F@HACC significantly reduced MDA content in roots (Figure 7E), with the 0.2 and 0.4 mg/mL groups exhibiting levels even lower than the uninfected control. This indicates the nanocarrier superior capacity to alleviate oxidative injury, likely due to its sustained release and rhizosphere‐targeted enrichment. In contrast, MDA levels in stems showed minimal variation among treatments (Figure 7F), while a slight increase was observed in leaves at higher doses (Figure 7G). This may reflect transient ROS fluctuations or localized hypersensitive responses triggered by systemic defense activation; nevertheless, MDA levels remained substantially lower than in untreated controls. POD, a key enzyme in H_2_O_2_ detoxification, exhibited a dose‐responsive trend. In roots (Figure 7H), even the lowest dose (0.05 mg/mL) significantly enhanced POD activity, suggesting early activation of H_2_O_2_‐scavenging pathways. Interestingly, POD activity decreased at higher concentrations (0.2 and 0.4 mg/mL), potentially due to negative feedback following reduced pathogen‐induced ROS stress. In stems (Figure 7I), POD activity was higher in the free PYR and control groups compared to nanocarrier treatments, indicating that PYR@PFC‐1‐F@HACC more effectively alleviated systemic oxidative stress. POD activity in leaves fluctuated (Figure 7J), showing lower values under high‐dose treatments, which aligns with constrained pathogen expansion and reduced ROS levels‐further supporting its efficacy in alleviating foliar stress. SOD, responsible for dismutation of superoxide radicals, was generally upregulated across all tissues. Root and stem SOD activity increased significantly in the 0.05 and 0.4 mg/mL groups (Figure 7K,L), indicating rapid and sustained activation of antioxidant defenses in response to initial pathogen stimulation. Leaf SOD activity also rose markedly (Figure 7M), with the greatest enhancement observed at 0.05 mg/mL, suggesting that even low concentrations of the nanocarrier system could improve fungicide targeting and stimulate systemic immunity.

Overall, the in planta result demonstrate dose‐dependent disease suppression by PYR@PFC‐1‐F@HACC, accompanied by reduced lipid peroxidation at the infection site and modulation of antioxidant enzyme activities, in line with the platform multi‐stimuli‐responsive release and support the protective mechanism inferred from the in vitro observations against *S. rolfsii*.

### 2.7 Eco‐Safety Assessment via Multi‐Level Biological Models

**FIGURE 8:**
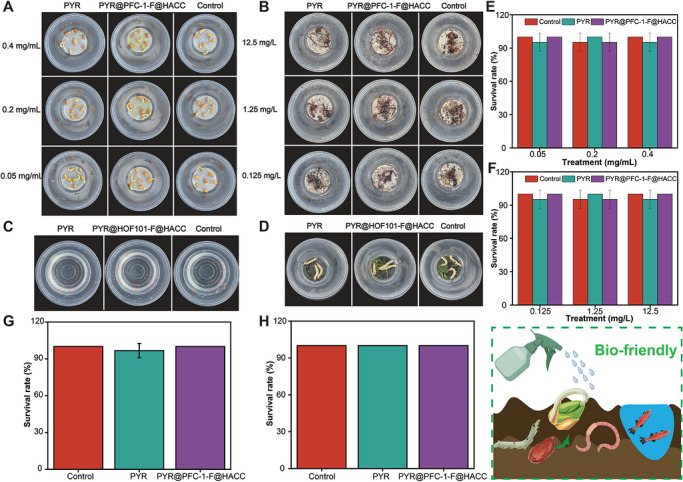
Biosafety and agro‐compatibility of PYR@PFC‐1‐F@HACC. (A) Peanut seed germination after 7 days under water (Control), free PYR, or PYR@PFC‐1‐F@HACC at 0.05, 0.2, 0.4 mg/mL (dose as PYR‐equivalent). (B) Earthworm assays after 48 h exposure to 0.125, 1.25, 12.5 mg/L (OECD‐based conditions). (C) Zebrafish immersion for 96 h at 12.5 mg/L. (D) Silkworm dietary exposure for 72 h at 0.125 mg/L. (E–H) Quantification of germination/survival rates corresponding to (A–D). Data are means ± SD of triplicate experiments. Asterisks (*) indicate a significant difference between sample type and the control according to a Student's *t*‐test (*p < 0.05, **p < 0.01).

To establish environmental compatibility at application‐relevant doses, we implemented a four‐tier bioassay spanning plants, soil invertebrates, aquatic vertebrates, and beneficial insects, and evaluated acute toxicity as well as endpoints readouts under controlled conditions. We first probed plant safety using a peanut seed germination assay. Sterilized seeds were exposed for 7 days to 0.05, 0.2, 0.4 mg/mL and scored for germination together with early seedling indices (Figure 8A). No visible inhibition, deformation, or chlorosis was observed across treatments; germination ratios and seedling metrics were statistically indistinguishable from the water control, as quantified in Figure 8E, indicating compatibility of the nanocarrier during the most sensitive developmental window and supporting subsequent in planta delivery. To gauge potential effects on soil fauna, we conducted an OECD‐style acute earthworm contact test. Worms were exposed for 48 h to suspensions containing 0.125, 1.25, and 12.5 mg/L suspensions (Figure 8B). Survival remained ≥95% with no abnormal surfacing, coiling, or hypoactivity; summary statistics in Figure 8F show no significant differences compared with the control. A mild downward trend for free PYR at the highest dose did not reach significance, whereas the nanocarrier remained comparable to the control across concentrations. Acute aquatic compatibility was assessed by immersing zebrafish at 12.5 mg/L for 96 h and monitoring mortality, erratic swimming, surface gasping, and external lesions (Figure 8C). No adverse behavior or external damage was detected, and survival matched the control (Figure 8G), consistent with low acute aquatic toxicity of the formulation at its working concentration. Potential impacts on beneficial terrestrial insects were examined using a silkworm dietary exposure. Larvae received 0.125 mg/L for 72 h (Figure 8D). Feeding, locomotion, and gross morphology remained normal; survival was comparable to control (Figure 8H), with no evidence of growth retardation or lethargy.

Taken together, Figure 8E‐H shows no significant deviations from control across plant, soil, aquatic, and insect models at application‐relevant doses. We attribute this favorable safety profile to the combination of a metal‐free crystalline host and a biopolymer shell, which together afford a chemically stable, biointerface‐compatible carrier. These results provide an initial eco‐safety basis for translating PYR@PFC‐1‐F@HACC as a potentially green, field‐ready pesticide‐delivery platform.

## 3 Conclusion

We establish a multi‐stimuli‐responsive system (PYR@PFC‐1‐F@HACC) that couples structural order with context‐responsive release for precision crop protection. PFC‐1‐F provides fluorine‐rich channel for loading PYR (∼30 wt.%) and functions as in planta fluorescent marker. The composite exhibits multi‐stimuli responsiveness‐chitosanase, H_2_O_2_, and mildly acidic pH‐delivering accelerated release with consistently good Weibull fits and ∼86%–88% cumulative release within 48–120 h, while remaining quiescent near neutrality. Mechanistically, antifungal efficacy arises from drug‐mediated inhibition with membrane disruption, augmented by light/ROS‐associated oxidative stress; in planta, the platform elevates SOD/POD and lowers MDA levels, indicating host‐defence remodeling. Standard biosafety assays show no significant adverse effects on peanut seed germination, zebrafish, or earthworms at working doses. By integrating a fluorinated, metal‐free HOF core with a cationic biopolymer shell, this work provides a generalizable blueprint for smart, sustainable agrochemical delivery and lays an important practical groundwork for next‐generation, precision plant‐disease management.

## 4 Experimental Section

### 4.1 Preparation of PYR@PFC‐1‐F Nanocomposite

To load PYR into the HOF, 10 mg of PFC‐1‐F was dispersed in 3 mL of methanol under ultrasonication. Subsequently, 90 mg of PYR was added, and the mixture was left undisturbed at room temperature for 12 h. The resulting product was collected by centrifugation, washed with methanol to remove unencapsulated drug, and dried under vacuum to obtain the PYR@PFC‐1‐F composite.

### 4.2 HACC Coating of PYR@PFC‐1‐F

For the fabrication of the outer HACC coating, 10 mg of HACC was completely dissolved in 40 mL of deionized water. Subsequently, 5 mL of this solution was added dropwise into a dispersion containing 50 mg of PYR@PFC‐1‐F. The mixture was subjected to brief sonication (10 min), followed by orbital shaking for 30 min, and then allowed to stand undisturbed at room temperature for 12 h to facilitate electrostatic assembly. The resulting composite was isolated by centrifugation, thoroughly rinsed with water to remove excess polymer, and dried for further analysis.

### 4.3 Characterization Techniques

The surface morphology of the samples before and after drug loading was examined using scanning electron microscopy (SEM, SU8010, Hitachi, Japan) operated at an accelerating voltage of 3.0 kV. Transmission electron microscopy (TEM) images were acquired on a JEM‐2100F microscope (JEOL, Japan) under an accelerating voltage of 200 kV. Crystalline structure was analyzed by wide‐angle x‐ray powder diffraction (XRD) using a D8 Focus diffractometer (Bruker, Germany). Zeta potential measurements were performed using a Zetasizer Nano ZS90 analyzer (Brookhaven Instruments, USA) to evaluate the surface charge characteristics. Fourier‐transform infrared (FTIR) spectra were recorded with a Nicolet IS10 spectrometer (Thermo Scientific, USA) to identify functional groups and chemical interactions. UV–vis absorption spectra of the materials were measured on a UV‐2600 spectrophotometer (Shimadzu, Japan). Specific surface area and pore size distribution were determined using nitrogen adsorption–desorption isotherms measured by a Micromeritics ASAP 2000 instrument (USA). The Brunauer–Emmett–Teller (BET) method was used to calculate surface area. Thermal stability was evaluated by thermogravimetric analysis (TGA) using a Mettler Toledo TGA 2+ thermobalance (Mettler Toledo, Switzerland) under nitrogen flow, with a heating rate of 10°C/min from 25°C to 800°C.

### 4.4 Stimuli‐Responsive Release Studies

To evaluate enzyme, ROS, and pH responsiveness, the in vitro release of PYR from PYR@PFC‐1‐F@HACC was investigated under chitosanase (2, 10, and 50 U/mL), H_2_O_2_ solutions (100, 500, and 1000 µm) and pH (4.5, 5.5, and 6.5). Briefly, 10 mg of PYR@PFC‐1‐F@HACC was dispersed in 10 mL of the corresponding medium and transferred into a dialysis bag, which was then immersed in 300 mL of the same medium at 25°C under gentle shaking (100 rpm). At predetermined time points, 2 mL of the external medium was withdrawn and replaced with an equal volume of fresh medium. The amount of released PYR was quantified by UV–vis spectrophotometry at 275 nm.

### 4.5 Translocation of PFC‐1‐F in Peanut

To evaluate the passive uptake and in planta distribution of the nanocarrier, bare PFC‐1‐F was applied onto the leaf surfaces of peanut plants via foliar spraying. At 12 and 24 h post‐application, treated plants were sampled, and tissues from leaves, stems, and roots were excised and rinsed thoroughly with deionized water to remove residual surface material. Samples were directly imaged under a confocal laser scanning microscope (CLSM, Carl Zeiss).

### 4.6 Contact Angle Measurement

To assess the wettability and adhesion characteristics of the nanocarrier formulations on leaf surfaces, contact angle measurements were performed using a contact angle goniometer (JC2000D, Shanghai Zhongchen, China). A 5 µL droplet of each sample was gently placed onto the adaxial surface of freshly harvested peanut leaves. Images were captured immediately, and the static contact angle was calculated using standard image analysis software. All measurements were conducted at room temperature, and each test was repeated in triplicate using different leaf samples.

### 4.7 In Vitro Antifungal Assay

The antifungal activity against *S. rolfsii* was evaluated using a mycelial growth inhibition assay. PDA plates were supplemented with various concentrations of the nanocomposite formulation (0.01, 0.05, 0.1, 0.2, and 0.4 mg/mL). After inoculation with fungal plugs, the plates were incubated at 26°C for 3 days. Colony diameters were measured, and the inhibition rate was calculated accordingly.

### 4.8 Live/Dead Staining and ROS Detection in Fungal Cells

To assess the viability of *S. rolfsii*, treated hyphae were stained using the Live/Dead viability kit (Thermo Fisher Scientific) and observed under a fluorescence microscope. Intracellular ROS accumulation was measured using DCFH‐DA as the fluorescent probe. Fungal samples were incubated with the probe in the dark for 30 min, washed, and analyzed by confocal laser scanning microscopy.

### 4.9 Detached Leaf Assay

To further evaluate the in vitro antifungal performance of the nanocomposite, a detached leaf assay was performed. Healthy peanut leaves were excised and placed adaxial side up on moist filter paper in Petri dishes. The PYR@PFC‐1‐F@HACC formulation was sprayed onto the leaf surfaces at concentrations of 0.05, 0.2, and 0.4 mg/mL. Treated leaves were incubated under controlled conditions (25°C, 70% humidity) with a 12 h light/12 h dark photoperiod. Photographs were taken at 6 and 12 days post‐treatment to visually assess disease progression and surface colonization. Moisture was maintained throughout the experiment to simulate natural infection conditions.

### 4.10 Plant Assay on Peanut

Peanut plants (*Arachis hypogaea L*.) were cultivated under controlled greenhouse conditions with regular watering and a 16 h light/8 h dark photoperiod. At the 3–4 leaf stage, plants were treated with PYR@PFC‐1‐F@HACC nanocomposite formulations via foliar spraying at a concentration of 0.4 mg/mL. After 6 days of treatment, samples from root, stem, and leaf tissues were collected for biochemical analysis, including antioxidant enzyme activity (SOD, POD), malondialdehyde (MDA) content, and protein quantification. All samples were immediately frozen in liquid nitrogen and stored at −80°C prior to analysis.

### 4.11 Biosafety Assessment

To comprehensively evaluate the environmental and biological safety of the nanocarrier system, phytotoxicity and acute toxicity tests were conducted on multiple representative non‐target organisms, including plants (peanut seeds), soil invertebrates (earthworms), aquatic species (zebrafish), and insects (silkworms). Please see Supporting Information for more detailed detection methods.

### 4.12 Statistical Analysis

Origin (OriginPro 2024b, OriginLab Corp., USA) was used for data plotting and curve fitting. GraphPad Prism 8.0 (GraphPad Software, Inc., USA) was used for data processing and visualization. Raw data were checked for completeness and screened for obvious outliers prior to analysis, no data transformation was applied unless stated otherwise. Results are presented as mean ± standard deviation (SD), and sample sizes are specified in the corresponding figure captions. Statistical analyses were performed using IBM SPSS Statistics 21.0 (IBM Corp., USA). The data were subjected to analysis of variance* (ANOVA), followed by Student's *t*‐test. Differences were considered statistically significant at *p* < 0.05.

## Author Contributions

**Guangming Ma**: conceptualization, investigation, writing – original draft, data curation, formal analysis, visualization, validation. **Huiyan Li**: validation, formal analysis. **Ying Zou**: conceptualization, writing – original draft, data curation, formal analysis, visualization, validation. **Xiaohong Pan**: writing – review and editing, writing – original draft, conceptualization, investigation, project administration. **Tianfu Liu**: conceptualization, writing – review and editing, project administration. **Jinlin Li**: validation, investigation. **Yao Wang**: validation, writing – original draft, data curation.

## Conflicts of Interest

The authors declare no conflicts of interest.

## Supporting information




**Supporting File**: advs75922‐sup‐0001‐SuppMat.docx.

## Data Availability

The data that support the findings of this study are available from the corresponding author upon reasonable request.

## References

[1] N. Lakshmi , A. Basha Shaik , P. Paramita Pal , et al., “Piperine, Reserpine and β‐Sitosterol Attenuate Stem Rot (Sclerotium rolfsii Sacc.) of Groundnut by Inducing the Secretion of Defense Enzymes and Phenolic Acids,” Chemistry & Biodiversity 19 (2022): 202100880. 10.1002/cbdv.202100880 35182415

[2] C. Shan , G. Wang , H. Wang , et al., “Assessing the Efficiency of UAV for Pesticide Application in Disease Management of Peanut Crop,” Pest Management Science 80 (2024): 4505–4515, 10.1002/ps.8155. 38703046

[3] M. R. Safari Motlagh , M. Farokhzad , B. Kaviani , and D. Kulus , “Endophytic Fungi as Potential Biocontrol Agents Against Sclerotium rolfsii Sacc.—The Causal Agent of Peanut White Stem Rot Disease,” Cells 11 (2022): 2643, 10.3390/cells11172643. 36078051 PMC9454559

[4] P. N. Meena , A. K. Meena , R. K. Tiwari , M. K. Lal , and R. Kumar , “Biological Control of Stem Rot of Groundnut Induced by Sclerotium rolfsii sacc,” Pathogens 13 (2024): 632, 10.3390/pathogens13080632. 39204233 PMC11357259

[5] A. Jogi , J. W. Kerry , T. B. Brenneman , J. H. Leebens‐Mack , and S. E. Gold , “Identification of Genes Differentially Expressed During Early Interactions Between the Stem Rot Fungus (Sclerotium rolfsii) and Peanut (Arachis hypogaea) Cultivars With Increasing Disease Resistance levels,” Microbiological Research 184 (2016): 1–12, 10.1016/j.micres.2015.11.003. 26856448

[6] D. G. Hirpara , H. P. Gajera , D. D. Savaliya , and R. V. Bhadani , “Characterization and Bioefficacy of Green Nanosilver Particles Derived From Fungicide‐Tolerant Tricho‐Fusant for Efficient Biocontrol of Stem Rot (Sclerotium rolfsii Sacc.) in Groundnut (Arachis hypogaea L.),” Journal of Microbiology 59 (2021): 1031–1043, 10.1007/s12275-021-1344-9. 34613606

[7] C. He , S. Liu , Y. Zhang , et al., “Multi‐Mechanism Encapsulation in a Bio‐Stimulant‐Based Smart Nanocarrier Enhances Fusarium Head Blight Control Through Synergistic Action and Triggered Release,” Advanced Functional Materials 35 (2025): 2506319, 10.1002/adfm.202506319.

[8] D. G. Hirpara and H. P. Gajera , “Molecular Heterozygosity and Genetic Exploitations of Trichoderma Inter‐Fusants Enhancing Tolerance to Fungicides and Mycoparasitism Against Sclerotium rolfsii Sacc,” Infection, Genetics and Evolution 66 (2018): 26–36, 10.1016/j.meegid.2018.09.005. 30219319

[9] D. B. Collinge , D. F. Jensen , M. Rabiey , S. Sarrocco , M. W. Shaw , and R. H. Shaw , “Biological Control of Plant Diseases—What Has Been Achieved and What Is the Direction?,” Plant Pathology 71 (2022): 1024–1047, 10.1111/ppa.13555.

[10] M. Nasrollahzadeh , S. M. Sajadi , M. Sajjadi , and Z. Issaabadi , Interface Science and Technology, ed. M. Nasrollahzadeh , S. M. Sajadi , M. Sajjadi , Z. Issaabadi , and M. Atarod , (Elsevier, 2019), 1–27.

[11] X. Jin , R. Xiao , Z. Cao , and X. Du , “Smart Controlled‐Release Nanopesticides Based on Metal–Organic Frameworks,” Chemical Communications 60 (2024): 6082–6092, 10.1039/D4CC01390E. 38813806

[12] C. Ma , G. Li , W. Xu , et al., “Recent Advances in Stimulus‐Responsive Nanocarriers for Pesticide Delivery,” Journal of Agricultural and Food Chemistry 72 (2024): 8906–8927. 10.1021/acs.jafc.4c00997 38602422

[13] Y. Wang , K. Ma , J. Bai , et al., “Chemically Engineered Porous Molecular Coatings as Reactive Oxygen Species Generators and Reservoirs for Long‐Lasting Self‐Cleaning Textiles,” Angewandte Chemie International Edition 61 (2022): 202115956, 10.1002/anie.202115956. 34931436

[14] J. Yin , J. Zhao , Z. Wang , et al., “Preparation of Multifunctional Nano‐Protectants for High‐Efficiency Green Control of Anthracnose,” Advanced Science 11 (2024): 2410585, 10.1002/advs.202410585. 39556712 PMC11672290

[15] X. Liu , G. Liu , T. Fu , et al., “Structural Design and Energy and Environmental Applications of Hydrogen‐Bonded Organic Frameworks: A Systematic Review,” Advanced Science 11 (2024): 2400101, 10.1002/advs.202400101. 38647267 PMC11165539

[16] A. M. Elewa , “Hydrogen‐Bonded Organic Frameworks (HOFs) From Design to Environmental Application,” Journal of Industrial and Engineering Chemistry 145 (2025): 169–190, 10.1016/j.jiec.2024.11.002.

[17] S. Chen , Y. Ju , H. Zhang , et al., “Photo Responsive Electron and Proton Conductivity Within a Hydrogen‐Bonded Organic Framework,” Angewandte Chemie International Edition 62 (2023): 202308418, 10.1002/anie.202308418. 37401627

[18] L. Tong , Y. Lin , X. Kou , et al., “Pore‐Environment‐Dependent Photoresponsive Oxidase‐Like Activity in Hydrogen‐Bonded Organic Frameworks,” Angewandte Chemie, International Edition 62 (2023): 202218661. 10.1002/anie.202218661 36719177

[19] Y. Liang , S. Wang , H. Jia , et al., “Pectin Functionalized Metal‐Organic Frameworks as Dual‐Stimuli‐Responsive Carriers to Improve the Pesticide Targeting and Reduce Environmental Risks,” Colloids and Surfaces B: Biointerfaces 219 (2022): 112796, 10.1016/j.colsurfb.2022.112796. 36063717

[20] Y. Liang , J. Song , H. Dong , et al., “Fabrication of pH‐Responsive Nanoparticles for High Efficiency Pyraclostrobin Delivery and Reducing Environmental Impact,” Science of The Total Environment 787 (2021): 147422, 10.1016/j.scitotenv.2021.147422. 33991920

[21] D. Nicodemo , F. E. Mingatto , A. de Carvalho , et al., “Pyraclostrobin Impairs Energetic Mitochondrial Metabolism and Productive Performance of Silkworm (Lepidoptera: Bombycidae) Caterpillars,” Journal of Economic Entomology 111 (2018): 1369–1375, 10.1093/jee/toy060. 29534200

[22] H. Zhang , Y. Zhang , M. Yu , et al., “Photo‐Responsive Supramolecular Hydrogels to Enhance Pesticide Bioavailability Through Multiple Structural Transformations,” Chemical Engineering Journal 505 (2025): 159473, 10.1016/j.cej.2025.159473.

[23] A. B. Tleuova , E. Wielogorska , V. S. S. L. P. Talluri , F. Štěpánek , C. T. Elliott , and D. O. Grigoriev , “Recent Advances and Remaining Barriers to Producing Novel Formulations of Fungicides for Safe and Sustainable Agriculture,” Journal of Controlled Release 326 (2020): 468–481, 10.1016/j.jconrel.2020.07.035. 32721524

[24] W. G. Birolli , B. F. da Silva , and E. Rodrigues‐Filho , “Biodegradation of the Fungicide Pyraclostrobin by Bacteria From Orange Cultivation Plots,” Science of The Total Environment 746 (2020): 140968, 10.1016/j.scitotenv.2020.140968. 32763599

[25] Y. Xie , X. Gong , Z. Jin , W. Xu , and K. Zhao , “Curcumin Encapsulation in Self‐Assembled Nanoparticles Based on Amphiphilic Palmitic Acid‐Grafted‐Quaternized Chitosan With Enhanced Cytotoxic, Antimicrobial and Antioxidant PROPERTIES,” International Journal of Biological Macromolecules 222 (2022): 2855–2867, 10.1016/j.ijbiomac.2022.10.064. 36240894

[26] J. Niu , C. Wang , K. Qiao , et al., “Quaternized Chitosan‐Based Organic‐Inorganic Nanohybrid Nanoparticles Loaded With prothioconazole for Efficient Management of Fungal Diseases With Minimal Environmental Impact,” International Journal of Biological Macromolecules 262 (2024): 129662, 10.1016/j.ijbiomac.2024.129662. 38266842

[27] L. Ma , M. Yu , Y. Ma , et al., “Ascendancy of Pyraclostrobin Nanocapsule Formulation Against Rhizoctonia solani: From a Perspective of Fungus,” Pesticide Biochemistry and Physiology 197 (2023): 105682, 10.1016/j.pestbp.2023.105682. 38072539

[28] Y. Mi , Y. Chen , W. Tan , J. Zhang , Q. Li , and Z. Guo , “The Influence of Bioactive Glyoxylate Bearing Schiff Base on Antifungal and Antioxidant Activities to Chitosan quaternary Ammonium salts,” Carbohydrate Polymers 278 (2022): 118970, 10.1016/j.carbpol.2021.118970. 34973785

[29] Y. Mi , J. Zhang , Y. Chen , et al., “New Synthetic Chitosan Derivatives Bearing Benzenoid/Heterocyclic Moieties With Enhanced Antioxidant and Antifungal Activities,” Carbohydrate Polymers 249 (2020): 116847, 10.1016/j.carbpol.2020.116847. 32933686

[30] H. Xie , G. Yu , X. Wang , et al., “Sea Urchin‐Like Nanocarrier for Enhancing Pesticide Retention and Rain Fastness on Hydrophobic and Hydrophilic Crop Foliage,” Chemical Engineering Journal 490 (2024): 151901, 10.1016/j.cej.2024.151901.

[31] J. Liu , Y. Wang , J. Chang , et al., “Laccase‐Responsive Biobased Nanocarriers From Lignin/Chitosan for Enhanced Foliar Adhesion and Targeted Pesticide Delivery,” ACS Sustainable Chemistry & Engineering 13 (2025): 18188–18201, 10.1021/acssuschemeng.5c07904.

[32] C. D. Georgiou , N. Patsoukis , I. Papapostolou , and G. Zervoudakis , “Sclerotial Metamorphosis in Filamentous Fungi Is Induced by Oxidative Stress,” Integrative and Comparative Biology 46 (2006): 691–712, 10.1093/icb/icj034. 21672779

[33] D. Liu , X. Mao , G. Zhang , et al., “Antifungal Activity and Mechanism of Physcion Against Sclerotium rolfsii , the Causal Agent of Peanut Southern Blight,” Journal of Agricultural and Food Chemistry 72 (2024): 15601–15612, 10.1021/acs.jafc.4c02519. 38950526

[34] I. Papapostolou , M. Sideri , and C. D. Georgiou , “Cell Proliferating and Differentiating Role of H_2_O_2_ in Sclerotium rolfsii and Sclerotinia sclerotiorum,” Microbiological Research 169 (2014): 527–532, 10.1016/j.micres.2013.12.002. 24388556

[35] S. Hu , L. L. Yang , C. Yan , et al., “pH and Redox Dual‐Responsive ZIF‐8‐Based Nanoplatform for Targeted Pathogens and Environmental Protection,” Chemical Engineering Journal 498 (2024): 154844, 10.1016/j.cej.2024.154844.

[36] B. Liang , S. Lu , J. Hu , J. Liu , and Y. Liu , “Green Nanopesticide: pH Response and Molybdenum Selenide Carrier With Photothermal Effect to Transport Prochloraz to Inhibit Sclerotinia Disease,” ACS Applied Materials & Interfaces 16 (2024): 15931–15945, 10.1021/acsami.4c00324. 38503698

[37] Y. Dong , T. Jiang , T. Wu , et al., “Enzyme‐Responsive Controlled‐Release Materials for Food Preservation and Crop Protection—A Review,” International Journal of Biological Macromolecules 254 (2024): 128051, 10.1016/j.ijbiomac.2023.128051. 37956811

[38] J. Zhou , G. Liu , Z. Guo , et al., “Stimuli‐Responsive Pesticide Carriers Based on Porous Nanomaterials: A Review,” Chemical Engineering Journal 455 (2023): 140167, 10.1016/j.cej.2022.140167.

[39] W. N. El‐Sayed , J. Alkabli , A. Aloqbi , and R. F. M. Elshaarawy , “Optimization Enzymatic Degradation of Chitosan Into Amphiphilic Chitooligosaccharides for Application in Mitigating Liver Steatosis and Cholesterol Regulation,” European Polymer Journal 153 (2021): 110507, 10.1016/j.eurpolymj.2021.110507.

[40] X. Ma , G. Wu , F. Dai , et al., “Chitosan/Polydopamine Layer by Layer Self‐Assembled Silk Fibroin Nanofibers for Biomedical Applications,” Carbohydrate Polymers 251 (2021): 117058, 10.1016/j.carbpol.2020.117058. 33142610

[41] Y. Ma , M. Yu , Y. Wang , et al., “A pH/Cellulase Dual Stimuli‐Responsive Cellulose‐Coated Metal–Organic Framework for Eco‐Friendly Fungicide Delivery,” Chemical Engineering Journal 462 (2023): 142190, 10.1016/j.cej.2023.142190.

[42] L. Han , X. Geng , J. Zhang , S. Sun , J. Li , and M. Zhao , “Cu‐Based Nanozymes Effectively Inhibit Proliferation of Fungal Pathogens by Catalyzing ROS Yield,” Materials Letters 355 (2024): 135509, 10.1016/j.matlet.2023.135509.

[43] Q. Yang , J. Yang , Y. Wang , et al., “Broad‐Spectrum Chemicals Block ROS Detoxification to Prevent Plant Fungal Invasion,” Current Biology 32 (2022): 3886–3897.e6, 10.1016/j.cub.2022.07.022. 35932761 PMC7613639

[44] X. T. He , Y. H. Luo , D. L. Hong , et al., “Atomically thin Nanoribbons by Exfoliation of Hydrogen‐Bonded Organic Frameworks for Drug Delivery,” ACS Applied Nano Materials 2 (2019): 2437–2445.

[45] Y. Zou , H. X. Liu , L. Cai , et al., “Strategy to Efficient Photodynamic Therapy for Antibacterium: Donor‐Acceptor Structure in Hydrogen‐Bonded Organic Framework,” Advanced Materials 36 (2024): 2406026, 10.1002/adma.202406026. 38923609

[46] M. Hefayathullah , S. Singh , V. Ganesan , and G. Maduraiveeran , “Metal‐Organic Frameworks for Biomedical Applications: A Review,” Advances in Colloid and Interface Science 331 (2024): 103210, 10.1016/j.cis.2024.103210. 38865745

[47] M. T. Khulood , U. S. Jijith , P. P. Naseef , S. M. Kallungal , V. S. Geetha , and K. Pramod , “Advances in Metal‐Organic Framework‐Based Drug Delivery Systems,” International Journal of Pharmaceutics 673 (2025): 125380, 10.1016/j.ijpharm.2025.125380. 39988215

[48] L. Sun , C. Hou , N. Wei , Y. Tan , Q. Liang , and J. Feng , “pH/Cellulase Dual Environmentally Responsive Nano‐Metal Organic Frameworks for Targeted Delivery of Pesticides and Improved Biosafety,” Chemical Engineering Journal 478 (2023): 147294, 10.1016/j.cej.2023.147294.

[49] M. Zhao , P. Li , H. Zhou , L. Hao , H. Chen , and X. Zhou , “pH/Redox Dual Responsive From Natural Polymer‐Based Nanoparticles for On‐Demand Delivery of Pesticides,” Chemical Engineering Journal 435 (2022): 134861, 10.1016/j.cej.2022.134861.

[50] T. Gao , B. Zhang , Z. Wu , et al., “Fabrication of ROS‐Responsive Nanoparticles by Modifying the Interior Pore‐Wall of Mesoporous Silica for Smart Delivery of Azoxystrobin,” Journal of Materials Chemistry B 11 (2023): 11496–11504, 10.1039/D3TB01954C. 37990572

[51] S. Yang , F. Lü , L. Wang , et al., “pH‐Responsive Metal–Organic Framework for Targeted Delivery of Fungicide, Release Behavior, and Sustainable Plant Protection,” Molecules 29 (2024): 5330, 10.3390/molecules29225330. 39598719 PMC11596698

[52] K. C. Paradva and S. Kalla , “Nanopesticides: A Review on Current Research and Future Perspective,” ChemistrySelect 8 (2023): 202300756.

[53] P. Dutta , A. Kumari , M. Mahanta , et al., “Nanotechnological Approaches for Management of Soil‐Borne Plant Pathogens,” Frontiers in Plant Science 14 (2023): 1136233, 10.3389/fpls.2023.1136233. 36875565 PMC9981975

[54] Z. Bao , Y. Wu , R. Song , et al., “The Simple Strategy to Improve Pesticide Bioavailability and Minimize Environmental Risk by Effective and Ecofriendly Surfactants,” Science of The Total Environment 851 (2022): 158169, 10.1016/j.scitotenv.2022.158169. 35995160

[55] C. Zhang , C. Tang , Q. Wang , Y. Su , and Q. Zhang , “Synergistic Effects of Oligochitosan and Pyraclostrobin in Controlling Leaf Spot Disease in Pseudostellaria heterophylla,” Antibiotics 13 (2024): 128, 10.3390/antibiotics13020128. 38391514 PMC10886130

[56] R. G. Alscher , N. Erturk , and L. S. Heath , “Role of Superoxide Dismutases (SODs) in Controlling Oxidative Stress in Plants,” Journal of Experimental Botany 53 (2002): 1331–1341, 10.1093/jexbot/53.372.1331. 11997379

[57] M. Morales and S. Munné‐Bosch , “Malondialdehyde: Facts and Artifacts,” Plant Physiology 180 (2019): 1246–1250, 10.1104/pp.19.00405. 31253746 PMC6752910

[58] S. Yang , D. Yan , M. Li , et al., “Ergosterol Depletion Under Bifonazole Treatment Induces Cell Membrane Damage and Triggers a ROS‐Mediated Mitochondrial Apoptosis in Penicillium Expansum,” Fungal Biology 126 (2022): 1–10, 10.1016/j.funbio.2021.09.002. 34930554

