# Supplementary material for: Multi‐Stimuli‐Responsive Hydrogen‐Bonded Organic Frameworks Nanocarriers Enable Targeted Fungicide Release and Plant Immune Regulation
Source: Adv Sci (Weinh). 2026 Jun 4;13(48):e75922. doi: 10.1002/advs.75922 (PMC13336423; doi:10.1002/advs.75922)
Supplement: Supplementary file 1 [file ADVS-13-e75922-s001.docx]

**Supporting Information**

**Multi-stimuli-responsive Hydrogen-Bonded Organic Frameworks Nanocarriers Enable Targeted Fungicide Release and Plant Immune Regulation**

*Guangming Ma^†1^, Ying Zou^†2^,* *Yao Wang^2^, Huiyan Li^1^, Jinlin Li^2^, Tianfu Liu^2^*, Xiaohong Pan^1^**

^1^State Key Laboratory of Agricultural and Forestry Biosecurity & Key Lab of Biopesticide and Chemical Biology, Ministry of Education & Ministerial and Provincial Joint Innovation Centre for Safety Production of Cross-Strait Crops, College of Plant Protection, Fujian Agriculture and Forestry University, Fuzhou, Fujian, 350002, P. R. China;

^2^ State Key Laboratory of Structural Chemistry, Fujian Institute of Research on the Structure of Matter, Chinese Academy of Sciences, Fuzhou, Fujian, 350002 P. R. China.

*Corresponding author.

E-mail address: [panxiaohong@163.com](mailto:panxiaohong@163.com) (Xiaohong Pan); [tfliu@fjirsm.ac.cn](mailto:tfliu@fjirsm.ac.cn) (Tianfu Liu)

^†^ The two authors contribute equally to this work.

**Contents**

[1. Synthetic routes and structures of target compounds H_4_TBAPy-F.	3](#_Toc225847265)

[2. Synthesis of PFC-1-F	6](#_Toc225847266)

[3. Experimental Section	6](#_Toc225847267)

[4. Experimental data	20](#_Toc225847268)

# 1. Synthetic routes of H_4_TBAPy-F

Scheme S1. Synthesis procedure for H_4_TBAPy-F.

**1.1 Synthesis of tetramethyl 4, 4', 4'', 4'''-(pyrene-1, 3, 6, 8-tetrayl)tetrakis(2-fluorobenzoate)**

A mixture of 3-fluoro-4-(methoxycarbonyl)phenylboronic acid (30.0 mmol), 1,3,6,8-tetrabromopyrene (5.0 mmol), K_2_CO_3_ (50.0 mmol), and Pd(PPh_3_)_4_ (0.25 mmol) was added to a biphasic system of toluene (250 mL) and water (25 mL) and stirred at 100°C under nitrogen atmosphere for 48h. Upon completion, the reaction mixture was poured into 250 mL of water and filtered to collect the resulting yellow solid. The crude product was washed thoroughly with ethyl acetate and dried in a vacuum oven at 80°C for 24 h to afford the target compound in 60% yield.
^1^H NMR (400 MHz, CDCl_3_): δ 8.18 (s, 4H), 8.17–8.13 (t, 4H), 8.00 (s, 2H), 7.52 (dd, 4H), 7.47 (dd, 4H), 4.01 (s, 12H) (Figure S1).

Figure S1 ^1^H NMR (400 MHz, CDCl_3_) spectrum of tetramethyl 4, 4', 4'', 4'''-(pyrene-1, 3, 6, 8-tetrayl)tetrakis(2-fluorobenzoate)

**1.2 Synthesis of 4, 4', 4'', 4'''-(pyrene-1, 3, 6, 8-tetrayl)tetrakis(2-fluorobenzoic acid) (H_4_TBAPy-F)**

The intermediate (2.8 g, 3.4 mmol) was dissolved in a mixture of tetrahydrofuran (THF, 100 mL) and methanol (100 mL), followed by the addition of 50 mL of 6 M KOH. The reaction mixture was refluxed overnight under stirring. Upon cooling to room temperature, the solvents were removed under reduced pressure. The resulting residue was dissolved in water and acidified with concentrated HCl in an ice-water bath to precipitate the product. The solid was collected by filtration, washed thoroughly with water, and dried under vacuum at 80 °C to afford the target compound as an off-white solid in near-quantitative yield.
¹H NMR (400 MHz, DMSO-*d*₆): δ 8.22 (s, 4H), 8.11 (s, 2H), 8.08–8.04 (t, 4H), 7.71 (dd, 4H), 7.64 (dd, 4H), 4.00 (s, 12H) (Figure S2).

Figure S2 ^1^H NMR (400 MHz, CDCl_3_) spectrum of 4, 4', 4'', 4'''-(pyrene-1, 3, 6, 8-tetrayl)tetrakis(2-fluorobenzoic acid

# **2.** **Synthesis of PFC-1-F**

H₄TBAPy-F (100 mg) was dissolved in 15 mL of DMF, followed by the addition of 100 mL of water. The mixture was stirred for 5 minutes, and then 100 mL of ethanol was added. The products were separated by centrifugation at 12,000 rpm for 15 minutes, and the resulting solid was further purified by washing with ethanol and acetone several times.

# 3. Experimental Section

**3.1 Photothermal effect testing**

The photothermal conversion efficiency (PTCE, *η*) of PFC-1 and PFC-1-F was evaluated based on previously reported methods. The efficiency was calculated using the following equation: methods, and was defined as

Equation S1:

$$\eta=\frac{hs({\Delta T}_{material}-{\Delta T}_{water})}{I(1-{10}^{-A})}$$

where I is the incident laser power, A is the absorbance of the aqueous suspension at the irradiation wavelength, and ΔT material and ΔT water represent the temperature elevation of the sample and pure water, respectively. The product hs, representing the heat transfer coefficient and surface area of the container, was determined from:

Equation S2:

$$hs=\frac{mc}{\tau_{s}}$$

where m is the mass of the aqueous solution (~3 g), c is the specific heat capacity of water (4.2 J g⁻¹ °C⁻¹), and τₛ is the system time constant, which was derived from the cooling stage using:

Equation S3:

$$t=-\tau_{s}ln(\theta)$$

where θ is a time dependent dimensionless parameter, known as the driving force temperature and defined as follows:

Equation S4:

$$\theta=\frac{T-T_{surr}}{T_{max}-T_{surr}}$$

where T is the instantaneous sample temperature, T_surr_ is the ambient temperature, and T_max_ is the maximum temperature reached during irradiation. All temperature readings were recorded using a digital thermal camera, and the calculations were repeated at least three times for reproducibility.

**Table S1.** **The parameters in the photothermal conversion rate formula**

| Sample | ΔT_material_-ΔT_H2O_ (^o^C) | T_max_ (^o^C) | Absorbance | τ_s_ | *η* (%) |
| --- | --- | --- | --- | --- | --- |
| PFC-1-F | 5 | 37.2 | 1.395 | 834 | 16.0 |

**3.2 Photodynamic testing**

The generation of singlet oxygen (^1^O_2_) was assessed using 1,3-diphenylisobenzofuran (DPBF) as a chemical probe. Upon reaction with ^1^O_2_, DPBF is oxidized to 1,2-dibenzoylbenzene (DBB), accompanied by a marked decrease in its characteristic UV-vis absorbance, particularly at 416 nm. Therefore, the photodegradation rate of DPBF can be used as an indirect measure of ^1^O_2_ production.

In a typical experiment, 200 μL of DPBF solution (3 mg/8 mL in ethyl acetate) was added to 3 mL of photosensitizer solution (1 mg/8 mL in ethyl acetate) in a quartz cuvette. The mixture was incubated in the dark until the baseline absorbance stabilized, and the initial absorbance at 416 nm (A_0_) was recorded. The cuvette was then irradiated in an ice-water bath using a 300 W xenon lamp equipped with a 400 nm long-pass filter. After 1 minute of irradiation, the absorbance at 416 nm (A) was measured. This light–measurement cycle was repeated seven times in total. Due to the overlap between the DPBF absorption band (400–450 nm) and the excitation wavelength, partial self-degradation of DPBF may occur and was considered during data interpretation.

**3.3** **Weibull kinetic analysis of fungicide release**

The release kinetics were fitted using the Weibull model:

$$\frac{M_{t}}{M_{\infty}}=1-exp\left[ -\left( \frac{t}{\lambda} \right)^{\beta} \right]$$

M_t_ is the cumulative amount released at time $t$, $M_{\infty}$ is the releasable amount at infinite time, $\lambda$ is the scale parameter, and $\beta$ is the shape parameter. This model has been widely used for dissolution and controlled-release kinetics.

**3.4 Enzyme Extraction Procedure for SOD, POD, and MDA Assays**

**3.4.1 Sample Processing**

Fresh plant tissues (root, stem, or leaf, ~100 mg) or microbial samples (~5 × 10^6^ cells) were collected and transferred into sterile microcentrifuge tubes. For microbial samples, the cells were resuspended in 1 mL of extraction buffer and lysed via ultrasonication (200 W, 3 s on/10 s off, 30 cycles) on ice. For plant tissues, the samples were homogenized in 1 mL of precooled extraction buffer using a tissue grinder in an ice bath. All homogenates were centrifuged at 8000 g for 10 min at 4 °C, and the supernatants were collected and kept on ice for immediate enzymatic analysis.

**3.4.2 Superoxide Dismutase (SOD) Activity Assay**

SOD activity was measured using a WST-1 based colorimetric assay. Briefly, 3 μL of extracted sample was added to a reaction mixture containing 45 μL of Reagent I, 20 μL of Reagent II working solution, 3 μL of Reagent III, 70 μL of distilled water, and 10 μL of Reagent IV working solution in a 96-well plate or EP tube. For each sample, a parallel control reaction was prepared with Reagent II in place of the sample, and blank tubes were included for background correction. After thorough mixing, the reaction mixtures were incubated at 37 °C for 30 minutes. Absorbance at 560 nm was recorded using a microplate reader. Inhibition rate was calculated as:

$$Inhibition rate \left( \% \right)=\frac{\Delta A Blank-\Delta A Sample}{\Delta A}Blank\times100\%$$

SOD activity was expressed as U/g protein using the formula:

$$\mathrm{SOD}\left( U/{10}^{4}\mathrm{cell} \right)=10\times Inhibition rate\div(1-Inhibition rate)\times F\div N$$

$$\mathrm{SOD}\left( U/g weight \right)=10\times Inhibition rate\div(1-Inhibition rate)\div W\times F$$

W: sample weight, g; N: total number of cells or bacteria; F: sample dilution factor.

**3.4.3 Peroxidase (POD) Activity Assay**

POD activity was determined by monitoring absorbance changes at 470 nm. A reaction mixture was prepared containing 120 μL of Reagent I, 30 μL of Reagent II working solution, 30 μL of Reagent III, 60 μL of distilled water, and 20 μL of sample extract. After mixing, 200 μL of the reaction solution was immediately transferred into a 96-well plate, and absorbance at 470 nm was measured at 30 s (A_1_) and 90 s (A_2_). The absorbance difference (ΔA = A_2_ – A_1_) was used to calculate enzymatic activity. POD activity was expressed as U/g fresh weight using:

$$\mathrm{POD}\left( U/{10}^{4}\mathrm{cell} \right)=9800\times\Delta A\times F$$

$$\mathrm{POD}\left( U/g weight \right)=9800\times\Delta A\div W\times F$$

W: sample weight, g; F: sample dilution factor.

**3.4.4 Malondialdehyde (MDA) Content Assay**

MDA content was determined using the thiobarbituric acid (TBA) reaction. A 100 μL aliquot of the enzyme extract was mixed with 300 μL of MDA working reagent and 100 μL of Reagent II in a microcentrifuge tube. After sealing, the tubes were incubated in a boiling water bath at 100 °C for 60 min and rapidly cooled in an ice bath. Samples were centrifuged at 10,000 g for 10 min, and 200 μL of the clear supernatant was transferred to a 96-well plate. Absorbance at 532 nm and 600 nm was recorded, and the corrected absorbance (ΔA) was calculated as:

$\Delta A=\left( A_{532}Sample- A_{532}\mathrm{Blank} \right)-\left( A_{600}Sample- A_{600}\mathrm{Blank} \right)$

MDA content was expressed as nmol/g fresh weight:

$$\mathrm{MDA}\left( nmol/{10}^{6}\mathrm{cell} \right)=53.763\times\Delta A\div N\times F$$

$$\mathrm{MDA}\left( nmol/g weight \right)=53.763\times\Delta A\div W\times F$$

W: sample weight, g; N: total number of cells or bacteria; F: sample dilution factor.

**3.5 Protein Concentration Determination**

Protein concentrations in all enzymatic extracts were measured using the Micro BCA protein assay. Briefly, 20 μL of sample or BSA standard was mixed with 200 μL of Micro BCA working reagent in a 96 well plate. After incubation at 60°C for 60 minutes, absorbance was recorded at 562 nm. Protein concentrations were calculated based on a standard curve generated from serial dilutions of a 200 μg/mL BSA standard.

**3.6 Biosafety Assessment**

**3.6.1 Peanut Seed Germination Assay**

The potential phytotoxicity of the formulations was assessed by monitoring seed germination and early seedling development. Surface-sterilized peanut seeds were placed on moist filter paper in plastic trays (n = 7 seeds per tray) and treated with 5 mL of PYR or PYR@PFC-1-F@HACC solution at concentrations of 0.05, 0.2, and 0.4 mg/mL. The trays were maintained at 25 °C under dark conditions for 7 days. Germination rate, root length, and visible abnormalities were recorded and compared with a deionized water control.

**3.6.2 Earthworm Acute Toxicity Assay**

The acute toxicity test of earthworms was carried out using the filter paper exposure method according to the relevant OECD standards. First, the cut filter paper was placed in a disposable transparent plastic bowl with a length of 10 cm and a depth of 6 cm. Then, 2 mL of test solution (0.125, 1.25, or 12.5 mg/L of PYR or PYR@PFC-1-F@HACC solution was slowly dripped into the filter paper. Later, ten earthworms were then placed in each plastic bowl. To ensure humidity and sealing inside the bowls, the mouths of the plastic bowls were sealed with sealing film and a small hole was opened for ventilation. The plastic bowls were placed at (20 ± 1)°C, 75% humidity, protected from light, with a separate control group of deionized water, and the survival of the earthworms after 48 h of exposure was recorded. Earthworms that did not respond to mechanical stimuli were defined as dead, those that showed symptoms such as large amounts of yellow body fluid exudation were defined as poisoned, and those with no obvious symptoms were defined as normal.

**3.6.3 Zebrafish Toxicity Test**

Zebrafish were tested for acute toxicity using a hydrostatic bioassay without feeding during the test. PYR or PYR@PFC-1-F@HACC with a concentration of 12.5 mg/L in 0.5 L aqueous solution was co-cultured with 10 living fish. After that, we recorded the survival of zebrafish after 96 h. A deionized water control group was set up. If any zebrafish dies, it should be promptly removed. All procedures involving zebrafish were approved by the Ethics Review Committee of Fujian Agriculture and Forestry University (Approval No. PZCASFAFU24140).

**3.6.4 Silkworm Toxicity Test**

To assess the potential impact on beneficial non-target insects, the toxicity to Bombyx mori larvae was evaluated by feeding fresh mulberry leaves sprayed with 0.125 mg/L PYR or PYR@PFC-1-F@HACC solution. The treated leaves were air-dried before feeding, and larvae were reared in ventilated plastic containers under standard breeding conditions (25 ± 1°C, 70% relative humidity, natural photoperiod). Observations were conducted for 72 h to evaluate survival rates, feeding activity, and visible abnormalities. The control group was fed untreated leaves

# 4. Experimental data

Figure S3 XPS survey spectra of PFC-1-F and PYR@PFC-1-F@HACC

Figure S4 Zeta potential of *S. rolfsii* hyphal suspensions after co-incubation with different treatments. Data are shown as mean ± SD (n = 3).
